# Legal and professional implications of shared care: a case study in oral anticoagulation stroke prevention therapy

**DOI:** 10.1186/s12913-015-0756-9

**Published:** 2015-03-10

**Authors:** Jacqueline A Nicholls, Henry WW Potts, Bridget Coleman, David L Patterson

**Affiliations:** Centre of Health Informatics and Multiprofessional Education, Institute Epidemiology and Healthcare, University College, London, UK; Department of Pharmacy, Whittington Health, London, UK; Department of Cardiovascular Medicine, Whittington Health, London, UK

**Keywords:** Shared care, Professional practice, Law, Anti-coagulation, Stroke prevention

## Abstract

**Background:**

Policy initiatives and technological advances enable the use of integrated shared care models of healthcare delivery whereby the focus of care is moved from the hospital to the community, and also of models where patients take increasing responsibility for monitoring and treatment. Such shifts may or may be perceived to change professional roles and responsibilities with implications to the delivery of a professionally and legally acceptable standard of care. We focus on oral anticoagulation and stroke prevention therapy to examine some possible professional and legal implications of the increasing use of shared care.

**Methods:**

This paper sought to explore how changes in service delivery influence the discharge of professional responsibilities to patients receiving oral anti-coagulation therapy in the context of clinicians’ legal and professional duties. We used a case study of the implementation of a distributed care anti-coagulation service. Qualitative data were collected using complementary methods: participant observation, reflective journaling and legal analysis.

**Results:**

Concerns identified by this study included a fear of litigation among both hospital and community-based professionals, a reluctance to embrace an extended role, uncertainty among professionals about the extent of their responsibilities and associated difficulties around adequate exchanges of information. These concerns reflected uncertainty among professionals about the legal and professional scope of the duty of care they owed patients.

**Conclusion:**

The findings from this study emphasise the importance of clear role definition, communication and inter-agency cooperation for the successful implementation of a shared care service in which threats to professional and legal standards of care are minimised.

## Background

The rapidly increasing complexity of healthcare has necessarily been accompanied by a commensurate need for more collaboration and sharing of information between healthcare professionals and across healthcare settings. Increasingly, health policy initiatives encourage physicians to move care from the hospital setting closer to the patient as part of a response to the need to provide sustainable, well-coordinated care for people with long-term conditions.

The challenge such initiatives face is to provide clinical and service-level integration that focuses on how care can be better provided around the needs of individuals, especially where this care is being given by a number of different professionals and organisations [1]. High functioning inter-professional sharing and collaboration is essential to the success of integrated, healthcare delivery [2,3]. Yet, the difficulties of achieving and maintaining successful high-functioning teams are well documented. As Freeman et al. explain there is considerable variability in professionals’ understanding of what constitutes multiprofessional working especially in relation to communication and understanding of the professional role [2].

A facet of integrated care that has been somewhat neglected concerns the traditional way in which a patient is viewed as ‘belonging’ to the physician responsible for his/her care. This has meant that patients initially see the physician who entrusts others to provide various services whilst retaining overall responsibility for care. Indeed this is the position supported by the legal principle of the non-delegable duty of care. Yet this sits uneasily with many physicians and allied healthcare professionals as they strive to deliver high quality care within their designated professional scope whilst confining themselves to practicing only in areas in which they are competent to practice. As a result although integrated shared care initiatives are usually welcomed by patients, physicians and other health care professionals may be reluctant to participate due to uncertainty about what it might entail, how they fit into the integrated system and associated fear of increased liability and vulnerability to litigation [4,5]. Such concerns are understandable in the increasingly litigious context of healthcare.

As a case study we consider oral anticoagulation and stroke prevention therapy (OASPT). This is a mainstay of the prevention of stroke in an increasing number of ‘at risk’ patients, principally using warfarin. Oral anticoagulation and stroke prevention therapy aims to reduce the risk of a thromboembolic event by maintaining patients at the optimum level of anticoagulation without producing an unacceptable risk of haemorrhage. Although the provision of OASPT has traditionally been through hospital-based services the increase in clinical need [6-8] has led to the development of a variety of models for delivering care. This includes schemes in which patients may attend community pharmacies or nurse-led clinics at GP surgeries for the routine monitoring and adjustment of their OASPT or the patient may self-test or self-manage.

There have been many examples of shared care developed over the last decades. Some such as antenatal shared care have been in existence for many years and the roles and responsibilities have been well defined. In contrast, anticoagulant and stroke preventions services, which bear the characteristics of many long-term conditions, have risks and complexities which are very difficult to manage safely and require very different approaches. The particular issues that present major issues that challenge conventional approaches include: the narrow therapeutic range of the anticoagulant drugs; the effects of diet and other drugs such as antibiotics and analgesics on oral anticoagulant drugs. As a result the oral anticoagulant itself can cause harm and death. However the benefit in terms of preventing stroke is substantial but the medication must be taken on a regular basis, the population is usually elderly, and many patients have multiple co-morbidities with associated changing medication regimes and involvement of many different healthcare professionals.

Most of the emergent models in OASPT involve a shift of service delivery from a traditional hospital outpatient setting to community settings and many harness electronic support. Such models are premised on a fundamentally new patient experience that is unconstrained by familiar points of engagement with healthcare or traditional channels for delivering information or care. Expanding a hospital-based service into a community-based service requires health care professionals in both settings to assume different responsibilities for patient care and, at the same time, some patients may take a more active role in their care. Allied to these changes are potential changes to the risks to healthcare quality and, therefore, to the way in which professional practice should be defined. Further exploration of specific changes in service delivery is required to assess how well-founded these concerns are and to shed light on the potential risks and dimensions of liability raised by a shift from hospital to community-delivered and hospital-supported care.

This paper makes a preliminary attempt to explore how changes in the service delivery of an oral anticoagulation service affect the delivery of care, with reference to existing law.

## Methods

### Study site

The site of the service discussed in this paper is in North Central London (NCL). The service aims to improve patient access to safe and effective OASTP. Optimised prescription requires an individual drug regime to avoid both under-coagulation leading to the risk of stroke and over-coagulation leading to the risk of haemorrhage. The scheme relies on remote access to a computerised decision support system (DSS) developed by a consultant cardiologist, and an associated electronic health record. The DSS records the clotting characteristics of a patient’s blood, and is complemented by an electronic record that enables prescribers to have access to relevant and current information concerning the patient; this is particularly important in the context of ACT given multiple potential drug interactions. The use of computerised predictive packages means that the recommended doses are within the same limits of accuracy as those suggested by experienced doctors, so that the service can be delivered by an appropriately trained nurse or pharmacist. This development, together with the increasing availability of portable, reliable and easy-to-use coagulometers, facilitated the development of novel forms of service delivery.

This service is an established integrated care service that embraces hospital outpatient departments and over 35 community sites. Several different models of service delivery operate. In some cases, primary care staff – community pharmacists, GPs or practice nurses – run community-based clinics. In other cases, there is an outreach service whereby pharmacists or nurses from the base hospital, run community-based clinics. Other patients attend the hospital as outpatients. In terms of managing a patient’s coagulation status there are six key roles: patient education in the use of an anticoagulant; initiation of a patient onto warfarin; testing and monitoring a patient’s coagulation status; dose adjustment and prescribing. These roles may be shared variably by healthcare professionals in different service models. In some circumstances patients may take responsibility for self-testing or self-managing.

Key organisational features include a Clinical Governance Board that brings together professionals from different disciplines and multiple organisations: cardiology, haematology, hospital and community pharmacists, GPs, managers and commissioners, patient representatives, researchers and other experts. The service also provides compulsory, assessed training for anticoagulant practitioners running clinics and has explicit Clinical Standard Operating Procedures to which all delivery sites should perform.

#### Legal analysis

We carried out a legal analysis of issues that arose from a range of data collection activities. The legal analysis used a combination of rule-based reasoning based on the established principles of the law of negligence and precedent analysis where parallels might be drawn between the facts presented here and previously decided cases. A search of the literature was conducted using the following databases Westlaw, Medline, CINAHL, Lexis, and BAILII. Key search terms identified were standard of care, professional negligence, duty of care, breach, consent, delegation, service models, shared care, distributed care.

As a way of focusing the legal analysis, we created case vignettes. These are fictional to preserve anonymity, but are based on one or more real incidents and on concerns raised by staff. The vignettes and this paper generally are not intended to be statistically representative of adverse events, problems or other issues that arose in the service. Rather, the vignettes were created and selected to illustrate a range of issues we encountered.

### Data collection

The issues identified for the legal analysis arose in the context of a broader series of studies of the service beginning in 2006. Full details of the data collection methods used in those studies have previously been reported [9-12].

In particular, participant observation, reflective journaling and a review of selected cases which caused professional anxiety and concern and of cases involving adverse events were used to explore the professional concerns and approaches of team members to the distributed service. DP and BC work as clinicians in the service, seeing patients and supervising junior staff and JN is also part of the Clinical Governance Board providing a legal perspective.

Throughout this work and in the prior studies, we sought close co-operation between researchers and service staff in a spirit of the co-production of knowledge, [13] with research findings continuously fed back to the clinical and software teams. This co-production approach, we believe, better captures the reality of what happens in a complex clinical service, but we recognise that it can also bring biases. We do not purport to have necessarily captured all the implications of shared care or to be able to say how frequently different implications occur. Rather, we have used the data collection to produce a set of problems for the legal analysis.

#### Participant observation

Researchers were participant observers at meetings from 2006 onwards, both of a formal and an informal nature, involving service staff (clinical and administrative) and/or researchers. Informal, unstructured conversations and interviews were carried out with the clinical team and other healthcare professionals in the local health ecosystem. Field notes were made as soon as possible after these.

The most significant formal meetings were of the Clinical Governance Board (CGB), which met regularly, approximately twice a year. Membership of the board is diverse, with those attending typically numbering around 20, and includes clinical (pharmacist, medical, nursing) and administrative service staff, clinical staff from other services, the software team, commissioners, researchers and patient representatives. All the authors have been on the board. Also significant were training and education events for anticoagulant practitioners. These were approximately biannual, and were also attended by patient representatives and members of the research team. They were larger than CGB meetings with around 70 attendees. The most significant informal meetings were between the clinical and software team. Where possible, meetings or conversations were held after significant events, e.g. negotiations around the service extending to a new site, a major critical incident or after CGB meetings.

We also had access to a range of documents which were analysed. These included: the Standard Operating Procedure for the service (in various different versions), materials considered at and minutes of the CGB, and Department of Health literature pertaining to anticoagulation.

#### Reflective journaling

JN and HP kept reflective journals over several years, during the course of the multiple studies mentioned, covering any issues that seemed pertinent. Issues were then discussed with the other authors on a regular basis.

#### Case studies creating concern among clinicians

In addition to discussing general concerns at the CGB meetings, professionals were actively encouraged by the consultant in charge of the service (DP) to share details of exemplar cases creating anxiety. Critical incidents were also identified in cooperation with the clinical team. Cases and incidents were initially reported by both primary and secondary healthcare professionals running the service either formally at the CGB meetings and/or directly to the Consultant in charge of the service or to members of the hospital-based service. The CGB meetings and informal discussions with various staff revealed some potential areas of concern. Additional research activities were specifically aimed at risk management [11-13] and also informed the work presented here.

#### Ethics

This study constituted an audit or service evaluation and therefore no ethics approval was needed from the NHS Research Ethics Committee, as discussed with the local R&D office. This study was exempt from approval by the UCL Ethics Committee as constituting an audit. We drew on a range of previous studies [9-12]: ethical approval was gained from the local research ethics committee for the collection of data which did not fall within the remit of service evaluation.

## Results and discussion

The vignettes presented here are informed by several years work within the oral anticoagulation and stroke prevention therapy service. They are not intended as results, rather it is hoped that they are recognizable as ‘real’ and typical of situations that arise in shared care. As these situations increasingly arise in shared care models of service delivery, what then are the potential medico-legal implications? The results are the legal analysis of the vignettes. We begin by considering how the principles of legal negligence might apply.

### The law of negligence

Professional responsibility – the legal basis governing professional duties and standards of care.

#### Duty of care - negligence and the scope of professional responsibility

Patients in the NHS expect the law to protect them from potential harm from negligent practitioners by establishing stringent requirements of high levels of professional practice. When things go wrong they expect legal consequences to attach to the health care professional. Furthermore, patients expect the law to embrace not only individual practitioners but the whole healthcare system so that individual components combine to optimise their health. Professionals’ concerns in relation to litigation are grounded in their appreciation of the legal duty of care which they owe to their patients which is, in turn, reinforced by professional Codes of Practice. This is complemented by a statutory duty of quality imposed on all Clinical Commissioning Groups (CCG) (and formerly on all NHS and Primary Care Trusts) by the Health & Social Care Act (2012) [14].

The law of negligence is the legal device used to give effect to the common law duty of care. It is based on the existence of a duty of care between two or more parties. When this duty is breached and the breach causes harm, a claim for negligence may arise. Most medical negligence claims are based on breach of the common law duty of care although if a patient is harmed through the negligent conduct of a healthcare professional, other consequences may follow, for example, rarely, criminal prosecution or professional disciplinary proceedings.

The existence of a duty of care is normally easily established. The basic rule is that you owe a duty of care to anyone you may foreseeably injure. Thus, hospital staff owe a duty of care to patients in hospital and GPs owe a duty of care to patients on their lists. In the context of this service, there are several potential categories of people who owe a duty of care to a patient:

- any of the involved medical professionals individually – cardiologists, haematologists, hospital pharmacists, community pharmacists, GP;
- any of the employers of those professionals, may also be vicariously liable, i.e. independent pharmacies, GPs, NHS Trusts, Clinical Commissioning Groups;
- the provider unit directly – CCG, independent pharmacies, GPs, NHS Trusts

Legal claims are most commonly brought against a Trust, which is legally responsible for the negligence of its employees either through the device of vicarious liability or as a result of a hospital’s non-delegable duty to its patients [15]. However, it is worth commenting further on the less commonly raised issue of direct liability referred to under (c) above. In this situation the claim would be that the providers themselves were negligent, for example, by failing to provide adequate numbers of suitably trained staff [16]. Thus, no individual professional might be held negligent but the provider itself could be. Other examples might be if there were no proper procedures to check that equipment was working properly or that staff were properly kept up to date with medical developments [17].

The usual test for negligence is the well-established *Bolam* test [18] which states that, ‘a doctor is not guilty of negligence if he has acted in accordance with a practice accepted as proper by a responsible body of medical men skilled in that particular art’. The test applies to all healthcare workers and despite much debate has been approved in the House of Lords [19-22]. Although the courts are yet to fully consider the question, it seems that when considering whether a trust has been directly negligent, the *Bolam* test for negligence will not apply i.e. a trust will not necessarily have a defence just because they are acting at a level other hospitals act at, if the judge decides those standards to be unreasonable [23]. Applying this to the outreach model(s) of OATSP service delivery suggests that the existence and use of distributed OATSP models elsewhere may, in itself, be unlikely to assist a provider unit in its defence of any claim, an issue which we found to be of concern to commissioners.

In relation to the hospital-based anticoagulation service it is self-evident that the consultant owes a legal and professional duty of care to all patients under the care of the service. It is equally uncontroversial that each of the participating healthcare professionals - nurses, pharmacists, GPs - owes a legal and professional duty of care to their patients. Similarly, in the outreach setting, all healthcare professionals, including the consultant in charge of the service, owe a legal and professional duty of care to their patients. A recurrent finding was that almost all community-based practitioners felt anxious about assuming this duty and unsure of its scope. In parallel, from the perspective of the consultant leading the service, the question that recurred was to what extent he remained liable for the actions of colleagues in the community. It is these issues which we consider next.

Legally, our starting point is that the duty of care is non-delegable [15], so a key question raised was, what is the extent of the consultant’s duty and how may this be met in practical terms? Similarly, what is the scope of the duty owed by GPs, nurse practitioners and community pharmacists? These concerns highlighted the need to explicate the duties, roles and responsibilities of other team members but how does current law assist us?

In relation to the delegation of care to community-based professionals, although required to ensure that the professionals to whom care is delegated are competent [24,25], the hospital consultant retains overall responsibility. In principle, delegation could be deemed negligent if, for example, the consultant failed to take reasonable steps to ensure that the delegees are appropriately qualified and trained. However, delegation is a two-way process and the professional delegee, be they GP, nurse or pharmacist, has a professionally imposed duty to practice only within spheres in which they are competent. Hence, if an instruction seems blatantly wrong, it is incumbent on a health care professional not to follow it, a position which was confirmed by two cases in which a pharmacist who failed to seek confirmation of a prescription that was patently wrong was found to be negligent [26,27].

To address the legal and professional requirements to ensure that delegees are adequately trained a central plank of this service is an assessed training programme that all practitioners are required to complete satisfactorily. The programme is subject to rigorous review and is complemented by ongoing follow-up procedures and audit checks. We found that the programme received overwhelmingly positive feedback from almost all participants. Yet, at the same time many of the same practitioners still voiced strong concerns about the scope of their responsibility and were reluctant to fully embrace their expanded role.

### Shared care: a series of case vignettes

In order to explore the dimensions of the concerns raised we will use a series of case vignettes. These are constructed case studies based on an amalgam of real life situations which were reported by professionals within NCL as being a source of concern.

#### Case vignette - shared care - unrelated medical admission to hospital – community interaction

*John is a 72 year old retired civil servant. He has active cancer and has suffered recurrent pulmonary emboli and so needs ongoing warfarin therapy. He has been taking warfarin for several years and is managed by the community service. He was admitted to hospital for unrelated reasons. He was discharged from hospital but no discharge summary was provided. The community service received a letter from a recent out*-*patient appointment indicating only that ‘the hospital’ had stopped the patient’s warfarin when John was an in-patient. Telephone calls to the hospital failed to clarify the situation meaning that the community service had to resort to asking the patient for details of his medication status.*

John’s case highlights the risks that present when there is inadequate communication between hospital and community providers. Clearly the hospital has a legal and professional responsibility to provide adequate discharge information to ensure that John is discharged safely. But John’s case raises interesting questions: how far should the community service be expected to go in tracking information? whose responsibility is it to ensure continuity of care of his warfarin management and how acceptable is it to be relying on a patient’s report of medication changes?

#### Case vignette – shared care with social services involvement

*Eleanor is a lady in her eighties with mild dementia, COPD and recurrent PE. Her mobility is limited and she requires constant portable oxygen. She is visited by the community anti-coagulation service every 2*-*3 weeks. Following hospital admission for a chest infection she was discharged without informing the GP or any of the community staff. A week post-discharge, when the healthcare assistant visited to do a routine check, Eleanor was found to be under-coagulated. She was sure that the hospital had taken her off warfarin and told her not to take any more. She was unsure which ward she was in and could not remember the names of any of the staff. The GP was unaware of the situation as no discharge summary had been provided and there was no written record. The community pharmacist contacted the hospital and, following several frustrating attempts to obtain the correct information, the hospital said that the patient’s warfarin had not in fact been stopped but that no discharge summary had been sent because ‘pharmacy had not approved it’.*

Eleanor’s case provides a further illustration of the difficulties that can ensue when information isn’t shared between all those with responsibility for a patient’s care. Eleanor’s situation was further confounded by the involvement of a healthcare assistant whose role, by definition, meant that she was unable to adjust Eleanor’s warfarin dose. Unlike John in our previous vignette, Eleanor was not able to assist the professionals involved, leading to inevitable frustration and anxiety reflected in this comment made by a nurse practitioner,

*‘I don’t know how we manage these sorts of incidents because where does the responsibility lie’*

#### Case vignette – GP failing to consider OASTP concerns/community OASTP axis

*William is a 69 year old ex-postman on warfarin who is described in medical notes as “not too stable” and suffers frequent nose bleeds. Following two particularly lengthy nosebleeds he telephoned the community anticoagulation service (ACS) who advised him to keep his warfarin dose unchanged. Over the subsequent 5 days William had some smaller nosebleeds and elected (without advice) to miss some of his doses of warfarin. His GP started him on an anti-infective for his nose bleeds which he had had before but did not counsel him in relation to warfarin, nor inform the ACS. The nosebleeds persisted and William continued to dose himself erratically. William attended the ACS and was found to be severely undercoagulated; he was advised to keep taking his warfarin and to inform the service of any further missed doses. The anticoagulation service wrote to the GP requesting a review of the appropriateness of warfarin for this patient bearing in mind any co-morbidities and his social situation, but received no reply and no indication that such a review had happened. Subsequently the GP admitted that he did not think he should ‘interfere’ with the warfarin.*

William’s case illustrates the threats to safety that can occur when different professionals do not operate from a shared, practical understanding of their respective roles. Combined with patients who may be panicked or frightened the potential for the standard of care to fall below what is acceptable is evident. The idea that by not ‘interfering’ a clinician somehow absolves him/herself of professional responsibility is clearly untenable. Despite this it was a recurring theme frequently occurring in the guise of clinicians not wanting to involve themselves in ‘specialist’ care even at the most basic level despite the risk to a patient of becoming seriously under or overcoagulated. In the risk context it is interesting to remind ourselves of the case of *Marriott v West Midlands HA* [28] in which a GP visited a patient who had suffered a fall and prescribed painkillers but did not suggest a full neurological examination as the risk of a brain clot was small. Despite the small risks the court deemed this to be negligent on the basis of the potential severity of the consequences to the patient if a clot existed.

#### Case vignettes – GP failing to consider the ‘over-responsible’ and ‘under-responsible’ patients

*Bob is an 83 year old retired driver who lives with his wife who is his main carer. He has been taking warfarin for atrial fibrillation for more than 5 years and is managed by the community ACS. Bob developed a chest infection and was seen by his GP at home. His GP observed an abdominal lump with surface bruising and provisionally diagnoses an abdominal haematoma so referred Bob to the hospital for a blood test and an ultrasound. However he did not notify the ACS nor discuss anti-coagulation with Bob. The ultrasound showed that Bob had two abdominal haematomas and, only at that stage, was he referred to the ACS for urgent review.*

It is unequivocal that a patient who presented to a GP with symptoms prompting a provisional diagnosis of suspected abdominal haematoma should have been referred to the OASTP service, rather than for a limited set of investigations which ignored their anticoagulation status. Bob’s case echoes the case of William as again the failure to refer Bob to the anticoagulation service in a timely fashion appeared to reflect the GP’s concern about ‘getting involved’, despite the fact that failure to refer at an appropriate time, bearing in mind the risks involved, has been regarded as evidence of negligence [28]. However, could it be argued that the relatively uncommon nature of a diagnosis of abdominal haematoma made it reasonable in a Bolam sense for the GP to act as he did in the first instance until a diagnosis was confirmed?

GP’s working outwith an anticoagulation service have, of course, always managed patients on warfarin. However, as both William’s and Bob’s cases illustrate, some GPs and other community professionals appeared uncertain as to how the system operated overall and behaved in ways which indicated a sub-optimal understanding of their role, leading to occasions when claims of sub-standard care would appear to be legitimate. Investigating these sorts of system failures further, we found that the Standard Operating Protocol was silent on the role that a GP working outwith this service should play in community-based OASTP service. This could be viewed as an oversight in relation to any claim for negligence on the part of the provider. Equally, the Service Specifications considered communications initiated by the patient or the anticoagulation practitioner but did not explicitly address the scenario of a GP needing to initiate contact, leaving the matter of when to instigate communication to an individual GP’s professional judgment.

#### Case vignettes – the ‘over-responsible’ and ‘under-responsible’ patients

*Edward is a retired molecular biologist who receives warfarin as part of the management of atrial fibrillation. His target INR is in the range 2-3. Edward has mild osteopenia and has fallen on two occasions. He is anxious about the possibility of fracturing his hip. He has extensively researched the scientific evidence concerning possible relationships between vitamin K levels and fracture and management of his stroke risk. As a result he has concluded that he wishes to reduce his target INR.*

*David is a 70 year old man who has taken warfarin for 5 years and has been self-testing for the last 2. As a result of becoming undercoagulated he has, in the past, suffered recurrent thrombi in his heart. Consequently the ACS sends him regular reminders to phone in his INR test results but he fails to do so unless something goes wrong.*

An essential part of warfarin therapy involves ensuring that patients are able to co-operate with their care. Whereas some patients struggle to understand their role, other patients are keen to manage their care themselves to the extent of becoming responsible for either self- management or self-testing. Although the move away from paternalistic models of care towards collaborative models in which decision-making is shared between patient and professional has been widely promoted by policy initiatives [29,30] we found many practitioners were very anxious about where responsibility lay when a patient was required to take more responsibility in practice. Edward and David reflect a growing contingent of informed patients who wish to be more involved in making clinical decisions about their care. When these decisions are contrary to the judgment of the clinician it raises the issue of where does legal liability fall. A strongly recurrent theme amongst clinicians was that that by responding to the clamour of voices urging for patients autonomy to be respected, they were caught in a no-win situation, making them responsible if things went wrong irrespective of the actions, however unwise, taken by a patient.

### Common issues

Overall, participants in this study expressed concern over the possibility of sub-standard care being offered and a profound fear of litigation. In broad terms, our findings fit into two overlapping categories: those related to professionals’ concerns about the scope of their role as individuals and those related to inter-professional and inter-agency communication across organisational boundaries.

Often patients on warfarin are admitted to hospital for an unrelated reason and we found some evidence of failure to provide adequate discharge information leading to a failure to ensure safe continuation of warfarin therapy on discharge from hospital. Safety is further undermined by uncertainty amongst the community professionals as to where responsibility lies. It is these sorts of situations that we suspect underlie the finding that GPs repeatedly reported, that of feeling undereducated in anticoagulation issues, despite receiving comprehensive training from the Hospital Trust. This concern may have arisen from an awareness among GPs that, legally, inexperience is no defence to failure to provide an acceptable standard of care [31,32]. A very real concern voiced by GPs appears to centre on concerns regarding failure to recognise the need for specialist support and being vulnerable to a claim of negligence for failing to refer appropriately. Under negligence law, the GP’s actions would be assessed by reference to the skills expected of a GP trained in oral anticoagulation therapy, not those of a cardiologist [33]. This makes the reluctance of the GPs more surprising as whereas a GP might not be deemed negligent in failing to know how to manage the warfarin s(he) could be deemed negligent for failing to refer the patient to the anti-coagulation service.

Shared care between hospital and community providers offers benefits to patients, but only if there is close collaboration and cooperation between the participating teams. As discussed above, this study revealed communication failures between different elements of the service, reflected at the patient and professional level by professional role ambiguity and failure to understand how different parts of the service fitted together. Hence, we found evidence of community practitioners failing to follow-up referrals to the hospital anticoagulation clinic and, conversely, some evidence of casual importance being accorded by hospital accident & emergency departments to ‘urgent’ information concerning a patient whose anti-coagulant status was volatile. More disappointingly, we found suggestion of a lack of co-operation at an organisational level resulting in, for example, evidence of insufficient information being transferred between hospital staff who considered that once they had discharged a patient they had no further responsibility to provide information to a patient’s GP about the patient’s anti-coagulation status.

The need to clarify roles in the system of delivering warfarin therapy has been highlighted previously [34]. Although, we could find no case law considering the legal liability of teams working across different geographical sites, the court has held that the way that a team of hospital doctors worked together produced a negligent level of care and in that case, the team leader was found to be negligent in having inadequately trained his team [35]. In relation to this service, it may be that the cross-site team-working could also provide a basis for a negligence action. The emphasis in clinical governance terms is on risk management at an individual clinician-patient level which highlighted a requirement for training to address the management of the risks of the wider team context within which the OASTP service is provided.

In many areas of tort law a claiming party who is found to have contributed in some way to their situation may be found to have been contributorily negligent and the remedies applied are adjusted appropriately to reflect this contribution. Traditionally, in healthcare law such a defence of contributory negligence by which some legal responsibility could be attributed to a patient has not been used. This reluctance presumably reflects the paternalistic model of clinician-patient relationship which has traditionally underpinned healthcare.

A successful legal claim for negligence requires not only that a duty of care is breached but also that the breach caused the harm suffered. In practice causation is tested by the ‘but for’ test so that ‘but for’ the alleged failure, the claiming party would not have suffered harm. In other words there is a direct link between an action and the harm suffered. On a paternalistic view of healthcare the main actor is the doctor or healthcare professional and the assumption is that the patient is adopting a passive ‘recipient’ role which absolves him of any real responsibility and, in a legal sense, means that his conduct cannot interfere with the chain of causation. It is curious that contributory negligence continues to be so rarely asserted. Yet, increasingly, as patients take more responsibility for their care it is questionable whether it remains appropriate to assume that patients’ actions cannot break the chain of causation.

In the UK we found only one clinical negligence case in which a successful defence was mounted on the basis of contributory negligence [36]. The case involved a patient whose cervical smear test was negligently reported as negative in 1988. In the following decade the patient was repeatedly advised to undergo further smear tests; she refused as she found the procedure painful and embarassing. She was diagnosed with cervical carcinoma in 1998 and claimed for negligence. Part of the defendant’s case was that the claimant’s behaviour had interfered with the chain of causation. The judge agreed and the claimant was found to be two thirds responsible. The basis for the judgment was largely pragmatic with little detailed judicial consideration of the underlying legal principles and so it is difficult for us to extrapolate to the situations faced by clinicians managing self-caring patients on warfarin.

## Conclusion

Extending a hospital-based anti-coagulation and stroke prevention service into different models of a community-based service requires health care professionals in both settings to assume different responsibilities for patient outcomes & quality of care. Consonant with this are potential changes to the risks to healthcare quality and to the way in which an acceptable standard of care is defined. These reflect a general problem of defining complex conduct prospectively, that of how to establish risk spreading and how to establish knowable standards of legal conduct. Alongside this problem remains the issue that physicians who are interested in promoting shared integrated care are concerned that shared responsibility with fellow professionals or patients does not at present mean shared liability. While these uncertainties remain, changes in ways of delivering services are likely to generate professional concerns about roles and responsibilities which may only partially be mitigated by stringent clinical governance procedures.
